# Poly[diaqua­(μ_2_-5-carboxy­pyridine-3-carboxyl­ato-κ^2^ *N*:*O* ^3^)hemi(μ_2_-oxalato-κ^4^ *O* ^1^,*O* ^2^:*O* ^1′^,*O* ^2′^)(μ_4_-pyridine-3,5-dicarboxyl­ato-κ^4^ *N*:*O* ^3^:*O* ^3′^:*O* ^5^)silver(I)terbium(III)]

**DOI:** 10.1107/S1600536809036393

**Published:** 2009-09-16

**Authors:** Hai-Fu Guo, Liang Qin, Xiang-Ying Hao

**Affiliations:** a School of Chemistry and Chemical Engineering, Zhao Qing University, Zhao Qing 526061, People’s Republic of China

## Abstract

In the title coordination polymer, [AgTb(C_7_H_3_NO_4_)(C_7_H_4_NO_4_)(C_2_O_4_)_0.5_(H_2_O)_2_]_*n*_, the Tb^III^ ion is eight-coordinated by three O atoms from three different pydc (H_2_pydc = pyridine-3,5-dicarboxylic acid) ligands, one O atom from one Hpydc ligand, two O atoms from one oxalate ligand and two water mol­ecules in a distorted square-anti­prismatic geometry. The Ag^I^ ion is coordinated in an almost linear fashion by two pyridyl N atoms from one pydc and one Hpydc ligand and has weak inter­actions with two carboxyl­ate O atoms. The carboxyl­ate groups of pydc and Hpydc ligands link Tb centers, forming a one-dimensional chain. The oxalate adopts a tetra­dentate bis-chelating coordination mode, connecting the chains into a two-dimensional layer. These layers are further assembled *via* [Ag(pydc)(Hpydc)] pillars and O—H⋯O and C—H⋯O hydrogen bonds into a three-dimensional coordination framework.

## Related literature

For general background to transition metal–lanthanide complexes, see: Barbour (2006 ▶); Kepert (2006 ▶); Kong *et al.* (2008 ▶); Rao *et al.* (2004 ▶); Wu *et al.* (2008 ▶); Zhang *et al.* (2005 ▶).
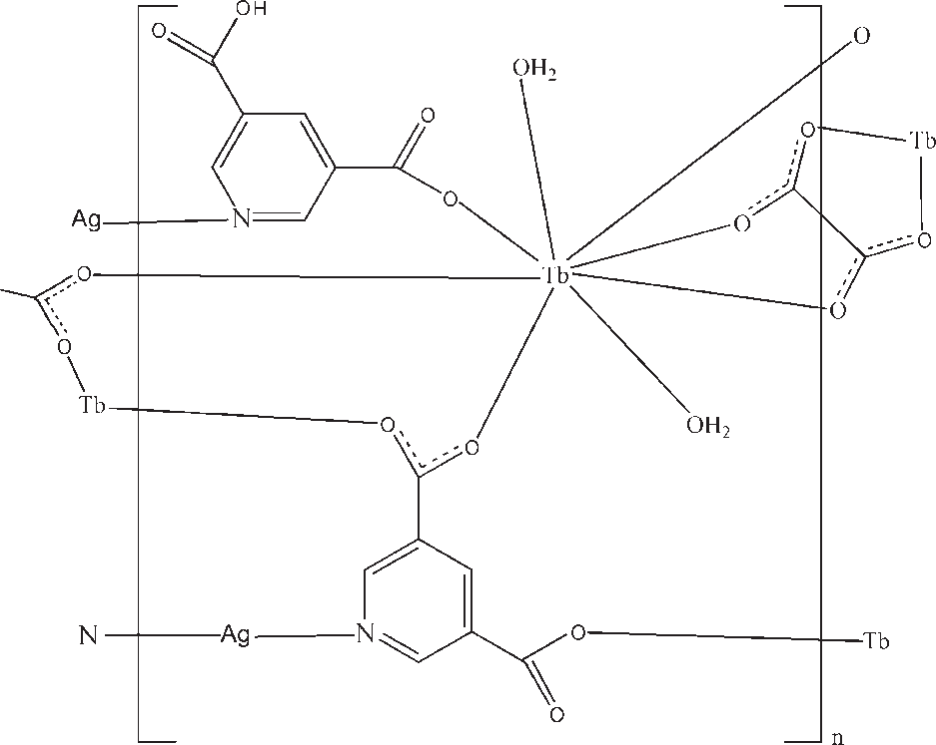


## Experimental

### 

#### Crystal data

[AgTb(C_7_H_3_NO_4_)(C_7_H_4_NO_4_)(C_2_O_4_)_0.5_(H_2_O)_2_]
*M* *_r_* = 678.05
Triclinic, 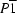

*a* = 7.592 (3) Å
*b* = 8.249 (3) Å
*c* = 14.241 (6) Å
α = 98.956 (4)°
β = 99.556 (4)°
γ = 95.839 (5)°
*V* = 861.3 (6) Å^3^
*Z* = 2
Mo *K*α radiation
μ = 5.29 mm^−1^
*T* = 293 K
0.30 × 0.24 × 0.19 mm

#### Data collection

Bruker APEXII CCD diffractometer
Absorption correction: multi-scan (*SADABS*; Sheldrick, 1996 ▶) *T*_min_ = 0.251, *T*_max_ = 0.378
4416 measured reflections
3032 independent reflections
2862 reflections with *I* > 2σ(*I*)
*R*_int_ = 0.018

#### Refinement

*R*[*F* ^2^ > 2σ(*F* ^2^)] = 0.023
*wR*(*F* ^2^) = 0.062
*S* = 1.09
3032 reflections
284 parameters
H atoms treated by a mixture of independent and constrained refinement
Δρ_max_ = 0.85 e Å^−3^
Δρ_min_ = −0.79 e Å^−3^

### 

Data collection: *APEX2* (Bruker, 2007 ▶); cell refinement: *SAINT* (Bruker, 2007 ▶); data reduction: *SAINT*; program(s) used to solve structure: *SHELXS97* (Sheldrick, 2008 ▶); program(s) used to refine structure: *SHELXL97* (Sheldrick, 2008 ▶); molecular graphics: *SHELXTL* (Sheldrick, 2008 ▶) and *DIAMOND* (Brandenburg, 1999 ▶); software used to prepare material for publication: *SHELXTL*.

## Supplementary Material

Crystal structure: contains datablocks I. DOI: 10.1107/S1600536809036393/hy2227sup1.cif

Structure factors: contains datablocks I. DOI: 10.1107/S1600536809036393/hy2227Isup2.hkl

Additional supplementary materials:  crystallographic information; 3D view; checkCIF report

## Figures and Tables

**Table 1:** Selected bond lengths (Å)

Tb1—O2	2.346 (3)
Tb1—O5	2.364 (3)
Tb1—O6^i^	2.365 (3)
Tb1—O8^ii^	2.317 (3)
Tb1—O9	2.444 (3)
Tb1—O10^iii^	2.401 (3)
Tb1—O1*W*	2.421 (3)
Tb1—O2*W*	2.468 (3)
Ag1—N1	2.172 (4)
Ag1—N2^iv^	2.162 (4)
Ag1—O7^v^	2.772 (3)
Ag1—O7^vi^	2.859 (3)

Symmetry codes: (i) 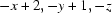; (ii) 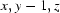; (iii) 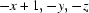; (iv) 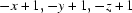; (v) 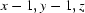; (vi) 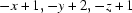.

**Table 2:** Hydrogen-bond geometry (Å, °)

*D*—H⋯*A*	*D*—H	H⋯*A*	*D*⋯*A*	*D*—H⋯*A*
O1*W*—H1*W*⋯O5^i^	0.84	2.08	2.820 (5)	147
O1*W*—H1*W*⋯O2*W*^i^	0.84	2.55	3.221 (5)	138
O1*W*—H2*W*⋯O9^vii^	0.84	2.05	2.855 (4)	159
O2*W*—H3*W*⋯O10^viii^	0.84	2.13	2.839 (4)	142
O2*W*—H4*W*⋯O1^viii^	0.84	2.04	2.873 (5)	173
O3—H3*A*⋯O1^ix^	0.90 (6)	1.71 (7)	2.554 (5)	154 (6)
C10—H10⋯O2*W*^ix^	0.93	2.40	3.314 (6)	169

Symmetry codes: (i) 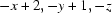; (vii) 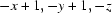; (viii) 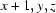; (ix) 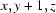.

**Fig. 1.**
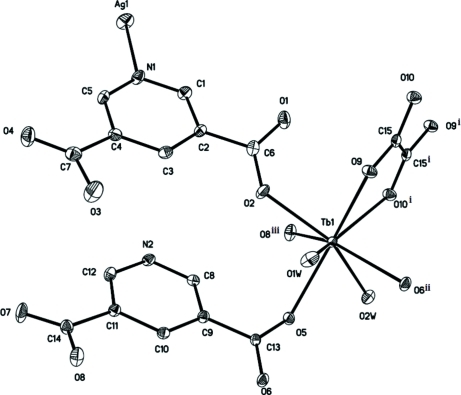
The asymmetric unit of the title compound. Non-H atoms are shown as 50% probability displacement ellipsoids. H atoms have been omitted for clarity. [Symmetry codes: (i) 1-x, -y, -z; (ii) 2-x, 1-y, -z; (iii) x, -1+y, z.]

**Fig. 2.**
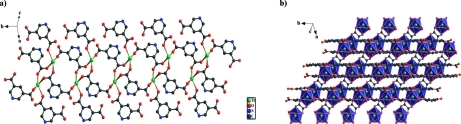
(a) A view of the one-dimensional chain in the title compound. (b) A polyhedral view of the two-dimensional layer. H atoms have been omitted for clarity.

**Fig. 3.**
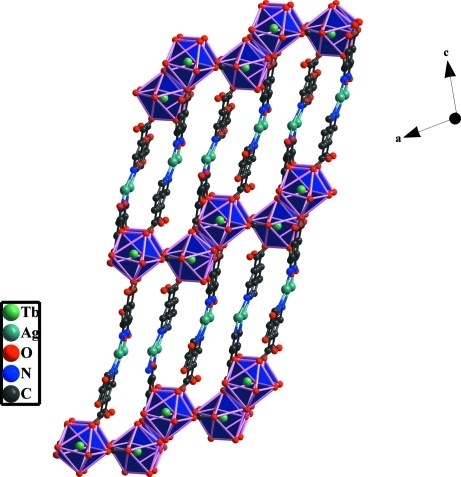
A polyhedral view of the three-dimensional framework. H atoms have been omitted for clarity.

## supplementary crystallographic information

### Comment

The design and construction of transition–lanthanide metal complexes has gained great recognition over the last decade because of their intriguing network topolopies and potential applications, and due to their magnetic properties, their capacity for gas storage, as luminescent materials, and so on (Barbour, 2006; Kepert, 2006; Kong *et al.*, 2008; Rao *et al.*, 2004; Zhang *et al.*, 2005). Pyridine-3,5-dicarboxylic acid (H_2_pydc) is a multifunctional bridging ligand possessing of O and N donors, which can thus be chosen to construct lanthanide–transition heterometallic complex *via* the carboxyl O atoms binding to lanthanides and N atoms bonding to transition metal ions such as Ag^I^ or Cu^I^ (Wu *et al.*, 2008). On the basis of above considerations, we utilize H_2_pydc, mixed 4d–4f metal ions and nitric acid as our building blocks. A new three-dimensional 4d–4f coordination framework resulted from the hydrothermal treatment of Tb_2_O_3_, AgNO_3_, oxalic acid, H_2_pydc and nitric acid in water.

As depicted in Fig. 1, the asymmtric unit of the title compound contains one Tb^III^ ion, one Ag^I^ ion, half an oxalate ligand, one pydc ligand, one Hpydc ligand and two water molecules. The Tb^III^ ion is eight-coordinated in a distorted square-antiprismatic coordination geometry by three O atoms from three different pydc ligands, one O atom from one Hpydc ligand, two O atoms from one oxalate ligand and two water molecules. The Ag^I^ ion is located in an almost linear configuration, defined by two N atoms from one pydc and one Hpydc ligands. The carboxylate groups of the pydc and Hpydc ligands link Tb^III^ center to form a one-dimensional chain with a shortest Tb···Tb distance of 5.261 (3) Å (Fig. 2a). The oxalate adopts tetradentate bischelating coordination mode to connect the neighboring chains into a two-dimensional layer (Fig. 2b). These layers are further assembled *via* [Ag(pydc)(Hpydc)] pillars into a three-dimensional coordination framework (Fig. 3). O—H···O and C—H···O hydrogen bonds (Table 1) involving the carboxyl group and coordinated water molecules enhance the stability of the three-dimensional network.

### Experimental

A mixture of Tb_2_O_3_ (0.183 g, 0.5 mmol), AgNO_3_ (0.169 g, 1 mmol), H_2_pydc (0.167 g, 1 mmol), oxalic acid (0.09 g, 1 mmol), HNO_3_ (0.12 ml) and H_2_O (10 ml) was placed in a 23 ml Teflon-lined reactor, which was heated to 443 K for 3 d and then cooled to room temperature at a rate of 10 K h^-1^. The colorless block crystals obtained were washed with water and dried in air (yield 46% based on Tb).

### Refinement

C-bound H atoms were placed at calculated positions and treated as riding on the parent C atoms, with C—H = 0.93 Å, and with *U*_iso_(H) = 1.2*U*_eq_(C). Water H atoms were tentatively located in difference Fourier maps and refined with distance restraints of O–H = 0.84 (1) and H···H = 1.39 (1) Å, and with *U*_iso_(H) = 1.5*U*_eq_(O). Carboxyl H (H3A) atom was refined isotropically.

### Crystal data

**Table:**

[AgTb(C_7_H_3_NO_4_)(C_7_H_4_NO_4_)(C_2_O_4_)_0.5_(H_2_O)_2_]	*Z* = 2
*M**_r_* = 678.05	*F*(000) = 646
Triclinic, *P*1	*D*_x_ = 2.615 Mg m^−^^3^
Hall symbol: -P 1	Mo *K*α radiation, λ = 0.71073 Å
*a* = 7.592 (3) Å	Cell parameters from 3600 reflections
*b* = 8.249 (3) Å	θ = 1.4–28°
*c* = 14.241 (6) Å	µ = 5.29 mm^−^^1^
α = 98.956 (4)°	*T* = 293 K
β = 99.556 (4)°	Block, colorless
γ = 95.839 (5)°	0.30 × 0.24 × 0.19 mm
*V* = 861.3 (6) Å^3^	

### Data collection

**Table:**

Bruker APEXII CCD diffractometer	3032 independent reflections
Radiation source: fine-focus sealed tube	2862 reflections with *I* > 2σ(*I*)
graphite	*R*_int_ = 0.018
φ and ω scans	θ_max_ = 25.2°, θ_min_ = 2.5°
Absorption correction: multi-scan (*SADABS*; Sheldrick, 1996)	*h* = −9→6
*T*_min_ = 0.251, *T*_max_ = 0.378	*k* = −9→9
4416 measured reflections	*l* = −16→17

### Refinement

**Table:**

Refinement on *F*^2^	Primary atom site location: structure-invariant direct methods
Least-squares matrix: full	Secondary atom site location: difference Fourier map
*R*[*F*^2^ > 2σ(*F*^2^)] = 0.023	Hydrogen site location: inferred from neighbouring sites
*wR*(*F*^2^) = 0.062	H atoms treated by a mixture of independent and constrained refinement
*S* = 1.09	*w* = 1/[σ^2^(*F*_o_^2^) + (0.0323*P*)^2^] where *P* = (*F*_o_^2^ + 2*F*_c_^2^)/3
3032 reflections	(Δ/σ)_max_ = 0.049
284 parameters	Δρ_max_ = 0.85 e Å^−^^3^
0 restraints	Δρ_min_ = −0.79 e Å^−^^3^

### Fractional atomic coordinates and isotropic or equivalent isotropic displacement parameters (Å^2^)

**Table:**

	*x*	*y*	*z*	*U*_iso_*/*U*_eq_	
Tb1	0.80756 (3)	0.29706 (2)	0.080761 (13)	0.01369 (8)	
Ag1	0.17209 (6)	0.40250 (5)	0.53284 (3)	0.03343 (12)	
O1	0.4372 (5)	0.2187 (4)	0.2009 (2)	0.0305 (8)	
O1W	0.7121 (5)	0.5291 (4)	0.0096 (2)	0.0278 (8)	
H1W	0.7742	0.5573	−0.0302	0.042*	
H2W	0.6316	0.5904	0.0165	0.042*	
O2	0.6205 (4)	0.4300 (4)	0.1747 (2)	0.0255 (7)	
O2W	1.0914 (4)	0.1769 (4)	0.0759 (2)	0.0253 (7)	
H3W	1.1070	0.1393	0.0200	0.038*	
H4W	1.1876	0.1905	0.1166	0.038*	
O3	0.4778 (6)	0.9748 (5)	0.2896 (3)	0.0449 (10)	
O4	0.3416 (5)	1.0334 (4)	0.4169 (2)	0.0346 (8)	
O5	1.0531 (4)	0.5114 (3)	0.1239 (2)	0.0203 (7)	
O9	0.5031 (4)	0.2064 (3)	−0.0119 (2)	0.0209 (7)	
N1	0.3034 (5)	0.5246 (4)	0.4335 (3)	0.0234 (8)	
N2	0.8987 (5)	0.7556 (4)	0.3678 (2)	0.0192 (8)	
C1	0.3576 (6)	0.4244 (5)	0.3627 (3)	0.0231 (10)	
H1	0.3377	0.3106	0.3601	0.028*	
C2	0.4420 (6)	0.4845 (5)	0.2934 (3)	0.0188 (9)	
C3	0.4629 (6)	0.6531 (5)	0.2942 (3)	0.0204 (9)	
H3	0.5162	0.6965	0.2476	0.025*	
C4	0.4032 (6)	0.7568 (5)	0.3654 (3)	0.0200 (9)	
C5	0.3273 (6)	0.6886 (5)	0.4347 (3)	0.0208 (10)	
H5	0.2917	0.7587	0.4838	0.025*	
C6	0.5041 (6)	0.3688 (5)	0.2177 (3)	0.0216 (10)	
C7	0.4047 (6)	0.9388 (6)	0.3621 (3)	0.0246 (10)	
C8	0.9565 (6)	0.6777 (5)	0.2901 (3)	0.0165 (9)	
H8	0.9658	0.5652	0.2849	0.020*	
C9	1.0023 (6)	0.7592 (5)	0.2182 (3)	0.0169 (9)	
C10	0.9849 (6)	0.9277 (5)	0.2249 (3)	0.0168 (9)	
H10	1.0142	0.9848	0.1770	0.020*	
C11	0.9234 (6)	1.0091 (5)	0.3038 (3)	0.0166 (9)	
C12	0.8845 (6)	0.9188 (5)	0.3740 (3)	0.0201 (9)	
H12	0.8469	0.9732	0.4279	0.024*	
C13	1.0667 (6)	0.6684 (5)	0.1333 (3)	0.0151 (9)	
C15	0.4376 (6)	0.0595 (5)	−0.0196 (3)	0.0168 (9)	
O6	1.1310 (4)	0.7500 (3)	0.0780 (2)	0.0211 (7)	
C14	0.9003 (6)	1.1915 (5)	0.3113 (3)	0.0202 (10)	
O7	0.8768 (5)	1.2673 (4)	0.3899 (2)	0.0328 (8)	
O10	0.2771 (4)	−0.0028 (3)	−0.0543 (2)	0.0219 (7)	
O8	0.9101 (5)	1.2520 (4)	0.2359 (2)	0.0262 (8)	
H3A	0.461 (9)	1.076 (8)	0.276 (4)	0.056 (18)*	

### Atomic displacement parameters (Å^2^)

**Table:**

	*U*^11^	*U*^22^	*U*^33^	*U*^12^	*U*^13^	*U*^23^
Tb1	0.01701 (13)	0.01146 (12)	0.01419 (12)	0.00272 (8)	0.00639 (8)	0.00282 (8)
Ag1	0.0419 (3)	0.0381 (2)	0.0290 (2)	0.00612 (19)	0.01825 (17)	0.01948 (17)
O1	0.049 (2)	0.0185 (16)	0.0251 (17)	0.0037 (16)	0.0116 (15)	0.0024 (13)
O1W	0.033 (2)	0.0227 (16)	0.0388 (19)	0.0149 (15)	0.0194 (16)	0.0180 (14)
O2	0.0281 (19)	0.0287 (17)	0.0241 (16)	0.0080 (15)	0.0150 (14)	0.0039 (13)
O2W	0.0241 (19)	0.0321 (17)	0.0215 (16)	0.0081 (15)	0.0070 (13)	0.0039 (13)
O3	0.067 (3)	0.0275 (19)	0.055 (2)	0.018 (2)	0.035 (2)	0.0209 (18)
O4	0.045 (2)	0.0241 (17)	0.0375 (19)	0.0126 (17)	0.0136 (17)	0.0024 (15)
O5	0.0284 (18)	0.0129 (14)	0.0208 (15)	0.0011 (13)	0.0096 (13)	0.0029 (11)
O9	0.0192 (17)	0.0147 (14)	0.0288 (16)	0.0013 (13)	0.0027 (13)	0.0063 (12)
N1	0.031 (2)	0.025 (2)	0.0207 (19)	0.0084 (17)	0.0136 (17)	0.0089 (15)
N2	0.022 (2)	0.0199 (18)	0.0191 (18)	0.0048 (16)	0.0083 (15)	0.0085 (14)
C1	0.027 (3)	0.019 (2)	0.028 (2)	0.005 (2)	0.011 (2)	0.0102 (18)
C2	0.019 (2)	0.023 (2)	0.016 (2)	0.0047 (19)	0.0063 (17)	0.0045 (17)
C3	0.020 (2)	0.023 (2)	0.020 (2)	0.0026 (19)	0.0065 (18)	0.0074 (17)
C4	0.016 (2)	0.017 (2)	0.027 (2)	0.0037 (18)	0.0038 (18)	0.0054 (17)
C5	0.022 (3)	0.025 (2)	0.017 (2)	0.0078 (19)	0.0061 (18)	0.0011 (17)
C6	0.027 (3)	0.022 (2)	0.017 (2)	0.008 (2)	0.0038 (19)	0.0046 (17)
C7	0.019 (3)	0.026 (2)	0.028 (2)	0.003 (2)	0.0013 (19)	0.008 (2)
C8	0.015 (2)	0.017 (2)	0.018 (2)	0.0021 (17)	0.0017 (17)	0.0056 (16)
C9	0.019 (2)	0.018 (2)	0.014 (2)	0.0030 (18)	0.0041 (17)	0.0033 (16)
C10	0.020 (2)	0.016 (2)	0.016 (2)	0.0018 (18)	0.0057 (17)	0.0056 (16)
C11	0.018 (2)	0.018 (2)	0.015 (2)	0.0034 (18)	0.0038 (17)	0.0042 (16)
C12	0.023 (3)	0.023 (2)	0.015 (2)	0.0060 (19)	0.0040 (18)	0.0027 (17)
C13	0.017 (2)	0.016 (2)	0.0119 (19)	0.0015 (17)	0.0021 (16)	0.0009 (16)
C15	0.021 (2)	0.015 (2)	0.014 (2)	0.0039 (19)	0.0058 (17)	0.0000 (16)
O6	0.0315 (19)	0.0181 (15)	0.0161 (14)	0.0030 (14)	0.0113 (13)	0.0034 (12)
C14	0.025 (3)	0.017 (2)	0.019 (2)	0.0035 (19)	0.0048 (18)	0.0037 (17)
O7	0.058 (3)	0.0231 (17)	0.0228 (17)	0.0110 (17)	0.0220 (16)	0.0011 (13)
O10	0.0198 (18)	0.0157 (14)	0.0303 (16)	0.0015 (13)	0.0053 (13)	0.0041 (12)
O8	0.045 (2)	0.0196 (15)	0.0179 (15)	0.0103 (15)	0.0109 (14)	0.0071 (12)

### Geometric parameters (Å, °)

**Table:**

Tb1—O2	2.346 (3)	N2—C8	1.352 (5)
Tb1—O5	2.364 (3)	N2—C12	1.351 (5)
Tb1—O6^i^	2.365 (3)	N2—Ag1^iv^	2.162 (4)
Tb1—O8^ii^	2.317 (3)	C1—C2	1.388 (6)
Tb1—O9	2.444 (3)	C1—H1	0.9300
Tb1—O10^iii^	2.401 (3)	C2—C3	1.382 (6)
Tb1—O1W	2.421 (3)	C2—C6	1.494 (6)
Tb1—O2W	2.468 (3)	C3—C4	1.388 (6)
Ag1—N1	2.172 (4)	C3—H3	0.9300
Ag1—N2^iv^	2.162 (4)	C4—C5	1.386 (6)
Ag1—O7^v^	2.772 (3)	C4—C7	1.508 (6)
Ag1—O7^vi^	2.859 (3)	C5—H5	0.9300
Ag1—Ag1^vii^	3.2867 (12)	C8—C9	1.381 (6)
O1—C6	1.260 (5)	C8—H8	0.9300
O1W—H1W	0.8401	C9—C10	1.400 (6)
O1W—H2W	0.8400	C9—C13	1.500 (6)
O2—C6	1.263 (6)	C10—C11	1.391 (6)
O2W—H3W	0.8402	C10—H10	0.9300
O2W—H4W	0.8400	C11—C12	1.388 (6)
O3—C7	1.309 (6)	C11—C14	1.522 (6)
O3—H3A	0.90 (6)	C12—H12	0.9300
O4—C7	1.207 (5)	C13—O6	1.241 (5)
O5—C13	1.273 (5)	C15—O10	1.260 (5)
O9—C15	1.244 (5)	C15—C15^iii^	1.535 (8)
N1—C5	1.343 (6)	C14—O7	1.244 (5)
N1—C1	1.348 (5)	C14—O8	1.262 (5)
			
O8^ii^—Tb1—O2	75.61 (11)	C8—N2—Ag1^iv^	114.8 (3)
O8^ii^—Tb1—O6^i^	141.96 (11)	C12—N2—Ag1^iv^	127.0 (3)
O2—Tb1—O6^i^	142.34 (10)	N1—C1—C2	122.6 (4)
O8^ii^—Tb1—O5	82.16 (11)	N1—C1—H1	118.7
O2—Tb1—O5	96.02 (11)	C2—C1—H1	118.7
O6^i^—Tb1—O5	89.03 (10)	C3—C2—C1	118.6 (4)
O8^ii^—Tb1—O10^iii^	81.37 (10)	C3—C2—C6	120.7 (4)
O2—Tb1—O10^iii^	109.81 (11)	C1—C2—C6	120.7 (4)
O6^i^—Tb1—O10^iii^	85.00 (10)	C2—C3—C4	119.1 (4)
O5—Tb1—O10^iii^	144.60 (10)	C2—C3—H3	120.5
O8^ii^—Tb1—O1W	135.70 (10)	C4—C3—H3	120.5
O2—Tb1—O1W	71.23 (11)	C5—C4—C3	119.0 (4)
O6^i^—Tb1—O1W	74.71 (10)	C5—C4—C7	120.2 (4)
O5—Tb1—O1W	73.14 (11)	C3—C4—C7	120.6 (4)
O10^iii^—Tb1—O1W	137.38 (11)	N1—C5—C4	122.3 (4)
O8^ii^—Tb1—O9	125.23 (12)	N1—C5—H5	118.9
O2—Tb1—O9	75.56 (11)	C4—C5—H5	118.9
O6^i^—Tb1—O9	79.71 (11)	O1—C6—O2	124.7 (4)
O5—Tb1—O9	146.28 (10)	O1—C6—C2	118.4 (4)
O10^iii^—Tb1—O9	66.38 (9)	O2—C6—C2	116.9 (4)
O1W—Tb1—O9	73.25 (11)	O4—C7—O3	126.2 (4)
O8^ii^—Tb1—O2W	73.67 (11)	O4—C7—C4	124.1 (4)
O2—Tb1—O2W	147.82 (10)	O3—C7—C4	109.6 (4)
O6^i^—Tb1—O2W	68.50 (10)	N2—C8—C9	122.3 (4)
O5—Tb1—O2W	70.60 (11)	N2—C8—H8	118.8
O10^iii^—Tb1—O2W	74.76 (11)	C9—C8—H8	118.8
O1W—Tb1—O2W	127.82 (11)	C8—C9—C10	118.9 (4)
O9—Tb1—O2W	131.32 (10)	C8—C9—C13	120.7 (4)
N2^iv^—Ag1—N1	164.83 (14)	C10—C9—C13	120.4 (4)
N2^iv^—Ag1—Ag1^vii^	108.80 (10)	C11—C10—C9	119.4 (4)
N1—Ag1—Ag1^vii^	86.10 (10)	C11—C10—H10	120.3
N2^iv^—Ag1—O7^v^	93.85 (11)	C9—C10—H10	120.3
N1—Ag1—O7^v^	92.40 (13)	C12—C11—C10	117.9 (4)
N1—Ag1—O7^vi^	83.19 (11)	C12—C11—C14	122.0 (4)
N2^iv^—Ag1—O7^vi^	107.77 (11)	C10—C11—C14	120.1 (4)
O7^v^—Ag1—O7^vi^	108.60 (9)	N2—C12—C11	123.2 (4)
Tb1—O1W—H1W	113.8	N2—C12—H12	118.4
Tb1—O1W—H2W	134.5	C11—C12—H12	118.4
H1W—O1W—H2W	111.6	O6—C13—O5	124.4 (4)
C6—O2—Tb1	129.6 (3)	O6—C13—C9	118.5 (3)
Tb1—O2W—H3W	114.5	O5—C13—C9	117.1 (4)
Tb1—O2W—H4W	131.1	O9—C15—O10	126.9 (4)
H3W—O2W—H4W	111.7	O9—C15—C15^iii^	117.3 (5)
C7—O3—H3A	113 (4)	O10—C15—C15^iii^	115.7 (4)
C13—O5—Tb1	133.9 (3)	C13—O6—Tb1^i^	137.7 (3)
C15—O9—Tb1	118.8 (3)	O7—C14—O8	126.2 (4)
C5—N1—C1	118.3 (4)	O7—C14—C11	118.2 (4)
C5—N1—Ag1	125.6 (3)	O8—C14—C11	115.6 (4)
C1—N1—Ag1	116.1 (3)	C15—O10—Tb1^iii^	120.5 (2)
C8—N2—C12	118.2 (4)	C14—O8—Tb1^viii^	155.4 (3)

Symmetry codes: (i) −*x*+2, −*y*+1, −*z*; (ii) *x*, *y*−1, *z*; (iii) −*x*+1, −*y*, −*z*; (iv) −*x*+1, −*y*+1, −*z*+1; (v) *x*−1, *y*−1, *z*; (vi) −*x*+1, −*y*+2, −*z*+1; (vii) −*x*, −*y*+1, −*z*+1; (viii) *x*, *y*+1, *z*.

### Hydrogen-bond geometry (Å, °)

**Table:**

*D*—H···*A*	*D*—H	H···*A*	*D*···*A*	*D*—H···*A*
O1W—H1W···O5^i^	0.84	2.08	2.820 (5)	147
O1W—H1W···O2W^i^	0.84	2.55	3.221 (5)	138
O1W—H2W···O9^ix^	0.84	2.05	2.855 (4)	159
O2W—H3W···O10^x^	0.84	2.13	2.839 (4)	142
O2W—H4W···O1^x^	0.84	2.04	2.873 (5)	173
O3—H3A···O1^viii^	0.90 (6)	1.71 (7)	2.554 (5)	154 (6)
C10—H10···O2W^viii^	0.93	2.40	3.314 (6)	169

Symmetry codes: (i) −*x*+2, −*y*+1, −*z*; (ix) −*x*+1, −*y*+1, −*z*; (x) *x*+1, *y*, *z*; (viii) *x*, *y*+1, *z*.
